# Holistic Context-Sensitivity for Run-Time Optimization of Flexible Manufacturing Systems

**DOI:** 10.3390/s17030455

**Published:** 2017-02-24

**Authors:** Sebastian Scholze, Jose Barata, Dragan Stokic

**Affiliations:** 1 Institut für angewandte Systemtechnik Bremen GmbH, 28359 Bremen, Germany; dragan@atb-bremen.de; 2 DEE-FCT, Universidade Nova de Lisboa, 2829-516 Caparica, Portugal; jab@uninova.pt

**Keywords:** context sensitivity, cyber-physical systems, sensors for context extraction, flexible manufacturing system, process optimization, self-learning systems, SOA

## Abstract

Highly flexible manufacturing systems require continuous run-time (self-) optimization of processes with respect to diverse parameters, e.g., efficiency, availability, energy consumption etc. A promising approach for achieving (self-) optimization in manufacturing systems is the usage of the context sensitivity approach based on data streaming from high amount of sensors and other data sources. Cyber-physical systems play an important role as sources of information to achieve context sensitivity. Cyber-physical systems can be seen as complex intelligent sensors providing data needed to identify the current context under which the manufacturing system is operating. In this paper, it is demonstrated how context sensitivity can be used to realize a holistic solution for (self-) optimization of discrete flexible manufacturing systems, by making use of cyber-physical systems integrated in manufacturing systems/processes. A generic approach for context sensitivity, based on self-learning algorithms, is proposed aiming at a various manufacturing systems. The new solution encompasses run-time context extractor and optimizer. Based on the self-learning module both context extraction and optimizer are continuously learning and improving their performance. The solution is following Service Oriented Architecture principles. The generic solution is developed and then applied to two very different manufacturing processes.

## 1. Introduction

The modern Flexible Manufacturing Systems (FMS) must deal with uncertainty; a change is expected, but the future is unknown [1]. The desire for ‘robustness’ stems from the fact that change is inevitable, both in reality and perception [2], and systems have to be continuously optimized to adapt to such changes. Both research and industrial communities have developed various approaches to cope with such changes. For example, the so-called changeable system method attempts to design the systems robust to various unknown changes [1]. Such systems need continuous run time optimization of various parameters, such as efficiency, energy consumption, availability, etc., and adapting to dynamically changing conditions under which they are operating. The classical approach to process optimization is to build specific off-line optimizations for different parameters and processes, each basing on different adaptive control laws. Building and, especially maintenance, of such solutions for highly dynamic FMS are time and costs consuming, and such solutions often cannot cope with many non-planned changes. The modern manufacturing system, with increased number of sensors, offer new opportunities for optimization of dynamically changing manufacturing processes. Especially Cyber-Physical Systems (CPS), which are being rapidly integrated in the current manufacturing systems, open new opportunities as complex, intelligent sensors providing enormous amounts of data on processes.

The objective of the presented research is to investigate how context sensitivity, using data from numerous sensors and CPS integrated in the modern manufacturing systems, can be used to realize a holistic solution for (self-) optimization of discrete flexible manufacturing systems. The context sensitivity allows for observation of changes in circumstances in which a system is operating, which in turn allows for a dynamic adaptation of the system to these varying conditions [3]. Thereby CPS play an important role as they offer new/additional sources of information, which can be used to achieve context sensitivity. Especially CPS directly integrated in manufacturing processes can be used for an effective identification of dynamically changing context under which the manufacturing system is operating. Self-learning capabilities are introduced enabling applicability of the solution to wide scope of manufacturing processes. The assumption is that building and adjustments of such generic context sensitive solution for various specific optimizations and processes is much more time/costs effective than building of classical optimizations solutions.

In this paper the applicability of the proposed holistic context sensitivity solution is demonstrated in two different application scenarios. The experiment in the first scenario investigates the potential optimization of energy consumption within a manufacturing process (secondary manufacturing process) by applying context sensitivity. The second experiment investigates the optimization of process control to gain a higher efficiency of the manufacturing process (prime manufacturing process) by applying context sensitivity as well.

The paper is organized in the following way: Section 2 provides the key research question and assumption, Section 3 includes a brief analysis of the state of the art in the key research areas relevant for the proposed solution. Section 4 and Section 5 describe the concept and implementation of the proposed solution. Section 6 provides the results of the experimental investigations of the solution, while Section 7 includes a brief analysis of the key benefits of the proposed solution, as well as the further research plans.

## 2. Research Question

The need for run-time optimizations of FMS is nowadays indispensable as described in the introduction of the paper. To achieve this there is a need for a solution that is capable of managing a high amount of data, complex models and algorithms. Furthermore, there is a need for a holistic solution that can be applied to different parameters, machines, systems and sectors. Thereby the effort for adjustments should be minimal. Such a holistic solution would prevent the building of scattered solutions and at the same time support the discovery of extensive problem solutions. However, in order to find such a generic solution, a common approach for the following two problems needs to be elaborated:

- Monitoring of changes during run-time within a FMS (e.g., changing process parameters, environment in which the system is operating etc.), which can be used for further processing.
- Extraction of a current context based on monitored data to be used for knowledge creation, which can be used for (self-) optimization of manufacturing processes.

### 2.1. Hypothesis

The generic context sensitive solution based on CPS, proposed in this paper, is easily adjustable to allow for optimization/adaptation of wide scopes of manufacturing systems. The context sensitivity allows for observation of changes in circumstances in which a manufacturing system is operating, which in turn allows for a dynamic adaptation of the system to these varying conditions [4].

### 2.2. Approach

The approach is based upon the assumption that the extracting of current context of the process allows for an effective self-optimization of manufacturing systems. Context extraction is a “generic observer”, which allows for run-time monitoring of processes and conditions, and extraction of knowledge about the changes in processes and conditions under which they are operating, i.e., context extraction seems to be an answer to both above listed problems and an effective way for run-time optimization of FMS. In this paper, the context is defined as “any information that can be used to characterize the situation of an entity” [5]. However, one of the key research challenges is which information should be used to describe the context (see Section 5) [6].

The approach, therefore, assumes that more efficient and re-usable embedded optimization services can be developed by using context sensitivity than by using classical optimization services. Such context aware services use information acquired from various sources (e.g., CPS, inputs of the human operator, etc.). The run-time context extraction uses a context model for device spaces.

Aiming to allow for application of the context model and context extraction services in different applications domains, the model consists of a generic model and a specific model, which instantiate generic concepts to allow for adjustments to the specific domain and application. The extracted context is used by embedded optimization services to adapt the process behavior to e.g., update process parameters. This work will investigate how such approach can be used as a generic solution to realize (self-) optimization of manufacturing systems.

## 3. State of the Art

### 3.1. Context Sensitivity and Context Modelling

Context Sensitivity is a concept propagated in the domains of Ambient Intelligence (AmI) and ubiquitous computing [7]. Existing research on context can be classified in two categories: context-based, proactive delivery of knowledge, and the capture and utilization of contextual knowledge. In the case of embedded services, the notion of context refers to process preferences of products and process skills of devices, physical capabilities of the equipment, environment conditions. As context integrates different knowledge sources and binds knowledge to the user to guarantee that the understanding is consistent, context modeling is extensively investigated within Knowledge Management research [8]. According to [9], context-sensitive computing uses contexts to provide relevant information and/or services to the users or applications. The relevancy depends mainly on the tasks or on the application domain [10].

The most challenging aspect of the application of the context sensitivity in the industrial processes is an effective acquisition/collection of data needed to extract a current context of the process. Therefore, the advanced sensors and CPS, as complex, intelligent sensors, are basic prerequisite for an effective application of this approach in manufacturing industry.

Key research task for the manufacturing domain for achieving context sensitivity is the definition of a generic and dynamic context model. Furthermore, the model need to be easy extendable for various manufacturing domains as well as for specific applications. The model must include the context of processes, equipment, products, humans and the use of knowledge for planning/executing various activities.

Some ontology-based solutions, concerned with the semantic representation of context and personalized service search and retrieval techniques, are described in [11,12]. There are also approaches to extend existing standards by adding/using context, such as KNX ISO [13]. The need to go beyond context representation to context reasoning, classification and dependency is also recognized [14]. Defining the context (model), that is required for achieving context sensitivity can be difficult, as indicated in [3,5]. Informal context models are often based on proprietary representation schemes without facilities for sharing the understanding about context between different systems [15]. Existing formal context models support formality and address a certain level of context reasoning [16]. Most common approaches to context-modelling are key-value models, Markup Scheme Models, Graphical Models such as Unified Modelling Language (UML), OOM, Logic- Based Models and Ontology-Based Models [17]. Some researchers [18] report about the comparison of different context modelling techniques. The present research on context modelling is often focused on ontologies [19]. This approach due to its easy extendibility and applicability for various processes seems to be the most appropriate for manufacturing industry. The modeling of context in the case of processes optimization in manufacturing industry is a challenging research task, as services in this domain are highly dynamic and reside in distributed environments [18].

In the presented paper ontologies are used for the modelling of context. Advantage of using ontologies it that the context can be modelled in a natural way and various reasoning mechanisms are available [20] that can be used for extraction of context. In addition, ontologies provide extendable mechanism, which are supporting the problem on how to infer high-level context information from low-level raw context data [21]. In [22,23] tool support for modelling of context as well as a selection of appropriate information sources are described that could foster the “easy” creation of context models.

### 3.2. Cyber-Physical Systems

According to [24] a Cyber-Physical Systems (CPS) is defined as an “integration of computation and physical processes”. The key idea is to combine the physical world (e.g., manufacturing process) with the virtual world (e.g., information processing). Thereby, CPS have a strong focus on a network of interacting CPS in order to achieve the desired functionality in contrast to traditional isolated systems. One example of a typical CPS is an intelligent manufacturing line, where the work of a machine is supported by the communication with its depending components.

The usage of CPS promises huge advantages against traditional systems. Hardware systems and software system can be interconnected arbitrarily. In addition, the connections of each other’s systems can be changed, deleted or newly built up on the fly. Furthermore, all accessible data, information and services can be deployed and utilized at any time anywhere in the system. Thus, cyber-physical systems’ services are independent from location, adapted to current systems requirements, partly autonomously, multifunctional and multimodal, networked and distributed along their application area [25,26].

It is expected that CPS will play an important role in future systems, especially also in manufacturing systems [27,28]. Recently there are several recommender models proposed to facilitate the sharing/extraction of knowledge [29]. The current Industry 4.0 initiative is strongly based on application of CPS in the manufacturing processes [30]. Although more and more CPS are applied in industrial environments and CPS are used in consumer environments as information sources for context sensitivity, this is still not investigated in current manufacturing systems.

### 3.3. Service Oriented Architectures

Service Oriented Architecture (SOA) is an approach that has been around since the 90s, when it was used in Tuxedo to describe “services” and “service processes” [31]. Service-orientation is still one of the most promising architectural designs for rapid integration of data and business processes. There are several standards available and accepted in industry that build on SOA principles, such as e.g., HTTP, JSON, XML, WS-*, etc. [22]. SOA is already heavily used in corporate and consumer environments but in embedded real-world environment SOA is emerging slower. The introduction of the OPC-UA architecture was a big step towards service-oriented architectures in industrial machine-2-machine sector. The upcoming trend to use CPS in industrial environments also fosters the usage of SOA-like principles in such environments. However, current research tries to apply SOA principles in domains, where such principles are not yet widely spread, such as industrial automation [32] or building automation [33] making it a promising approach for context aware solutions.

### 3.4. Self-Learning

Evolvable Production Systems are complex and lively composed of intelligent modules that interact, through bio-inspired mechanisms, to assure high system availability and seamless reconfigurations [34]. While the changeable system approach aims to design the systems robust to various unknown changes [1], self-learning production systems adapt themselves to changes based on learning in real time. Intensive research activities in the domain of self-learning systems have proved that the machine learning techniques, dynamic self-adaptation and operator’s feedback in the loop can be effectively applied in various systems to increase their intelligence and allow for adaptation to changeable conditions. In manufacturing systems in particular, these methods have been proven to be especially useful for monitoring/diagnosis [35,36]. However, the application of self-adaptation and self-learning of production systems based on context sensitivity in industrial practice is unexplored [37,38,39].

## 4. Concept for Context Sensitivity

**Figure 1:**
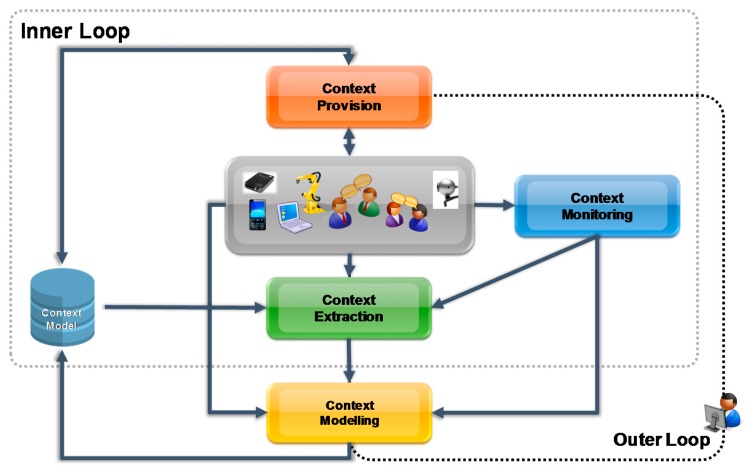
Context Sensitivity Concept.

The concept to achieve context sensitivity in FMS is shown in Figure 1. Context Monitoring is used to collect “raw data” from the FMS. The collected data is subsequently used by the Context Extraction to derive the current context of the FMS. In a next the current identified context is provided to upstream services (Context Provision). The provided context can for example used for generating knowledge about a manufacturing process. This knowledge will be used as a basis for operational decisions. This generated knowledge in turn forms the basis for decisions about optimizations of specific manufacturing processes. Decisions regarding the optimization of manufacturing process can be (a) short-term (specific tasks of a manufacturing process) and (b) long-term (overall manufacturing process).

As explained above, for achieving context sensitivity in production systems, CPS as well as other sensors and information sources are used to collect data about manufacturing process (as a sub-set of a whole production system). The collected data is used in the next step for identifying the current context of the monitored manufacturing processes. The identification starts through context monitoring services, which are, e.g., services for monitoring of processes or of a user interacting with a system for changing conditions [4]. The monitored “raw data” is transformed into a “standardized” data format by the monitoring services in order to allow further processing by the context extraction services. The context extraction service identifies current context by instantiating monitored data into the context model. Furthermore, reasoning techniques are used to support context identification. For reasoning, previously identified context and the context model is used, which is stored in the context repository. In contrast to many current approaches, where often only data about location/user is used for identifying context, the presented approach uses any monitored information that can be instantiated in the context model for identifying context. After the current context is identified, it is sent to the system adapter services, which are responsible for the system adaptation. In addition, the outer loop supports updating the context, based on the used concepts and relations of the identified context.

## 5. Implementation

**Figure 2:**
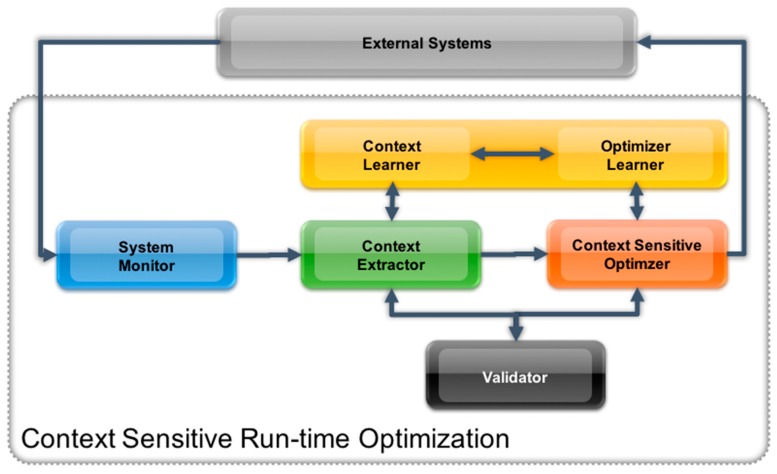
“Reference” Architecture to achieve context sensitivity for manufacturing systems.

Section 4 described how the context sensitivity can conceptually be used to allow for run-time optimizations of manufacturing systems. However, to allow for implementation of such a concept, an architecture is required, that can be integrated into existing manufacturing systems and allows to operate unobtrusively. To achieve such a “reference” architecture several application cases and scenarios from different industrial sectors have been analyzed. The key tasks performed by the components in this architecture are: monitor for contextual changes, identification of context, adaptation of system behavior and learning based on executed adaptations. The resulting architecture is depicted in Figure 2.

The components of the proposed system include:

- System Monitor, Context Extractor (including the Context Model) and Context Sensitive Optimizer—see Section 5.1, Section 5.2, Section 5.3 and Section 5.4 for detailed explanation of these services.
- Adaptation Learner and Context Learner: These services allow the system to learn. Key factor for the learning are the results of the Validator Services (operator’s feedback). These results are analyzed using data mining techniques and are used to improve the operation of the Context Extractor and the Context Sensitive Optimizer during run time (see also Section 5.4).
- Validator (for Context and Adaptation): These services are measuring the performance of optimization and context extraction. The measurement is either based on the manual feedback of the operator (e.g., acceptance of optimization proposals) or on statistical analysis in case the system operates in automatic mode. The results of the validator services are the key input for the learning services.

For simplicity, the Data Access Layer, Data Processing and the Service Infrastructure as well as supporting services/modules are not shown in Figure 2. However, the overall architecture is following a strict SOA approach.

### 5.1. Context Model

For the extraction of current context, an underlying model is required that supports the identification of context. In that sense the context model forms the basic data model that is used for the context identification/extraction. The proposed approach uses ontologies as technology for modelling the context model. Contrary to many ontologies, the context model proposed in this work is not foreseen, to define a full description of all possible context, but to model the concepts that are required for supporting the context identification [10].

The application of the proposed solution to a specific domain normally requires adjustment of the context model. Therefore, a general and extensible context model is proposed. It is in a format that meets several requirements: help to describe and capture context easily; help to manipulate context; facilitate context consumption by services. Therefore, the context model consists of a layered ontology approach. The model includes:

- the generic device context model
- the domain specific and/or
- application-specific context model(s).

**Figure 3:**
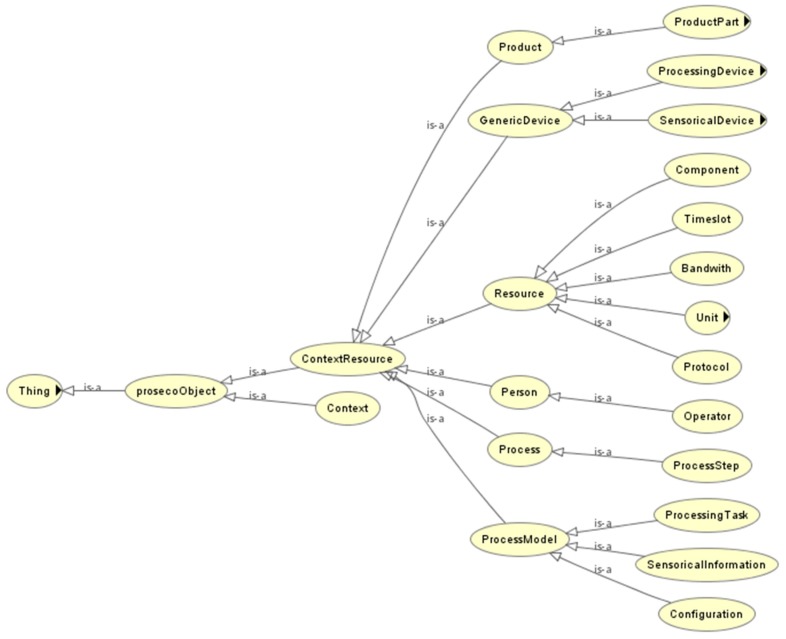
Context Model for device spaces.

The generic device context model defines the high-level context. The other layer(s) define the domain and/or application specific context model(s). The context model to be used in the proposed approach consists of all three layers. Thus, the context model is a semantic model for an integrated representation of machine, device and processing knowledge (including information of goal, activity, resource, etc.) as well as its generation. The model developed is defined as high-level structured representations of the product, processes and resources involved in process and their relationships. The generic context model defines concepts such as: Generic Device, Production Unit, Process Step, Operator, Resource, etc. Subsequent, the sector specific context model defines concepts such as: Manufacturing Process, NC controlled Lathe, Shoe Machine, etc. Finally, the Application Specific part contains specific products and processes (see Figure 3 for an excerpt of the above-mentioned context models).

The research is focused on development guidelines to effectively define context models for various applications [3]. Some basic principles for context modelling were elaborated and followed: (1) Description of context: In practice, it is virtually impossible to model all possible context information. An approach to create the full description of all possible context would be too time and cost intensive. Therefore, “only” the concepts should be modelled, that are relevant for the extraction of current context; (2) Availability of Context: In order to allow an efficient identification/extraction of context only context should be modelled that can be either provided by automatic monitoring by the system or manually provided by the human operator. However, provision of context information should be as easy as possible. Therefore, the context to be modelled should be (relatively) easy acquirable; (3) Cost of Context Modelling: Intuitively, if we could model as much context factors in as much details, the accuracy of context will be higher. At the same time the costs for the modelling of context are raising the more detailed the context is modelled. Therefore, it is important to find a good trade-off between investments due to context modelling and potential optimizations due to context extraction and adaptation.

### 5.2. Context System Monitor

The objective of the System Monitor is to receive raw sensor data and provide aggregated data. In order to monitor “raw sensory data”, the System Monitor need to be connected to diverse legacy systems, such as CPS, Web Services, file systems etc. To achieve this the System Monitor implements a Service-oriented configurable monitoring architecture (SOMA). Each system to be integrated into the System Monitor will be monitored by a specific monitor, that delivers its data to the superior Generic Monitor for further processing. The Generic Monitor is able to standardize and correlate data from specific systems for further processing in the Context Extractor module.

**Figure 4:**
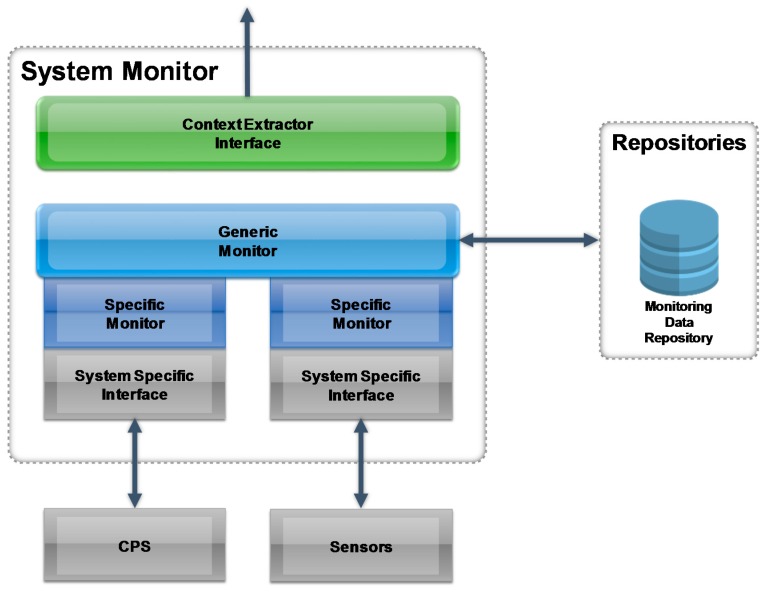
Context System Monitor.

The main component of the System Monitor is the modular monitoring process, used for all monitoring services with an extendable and configurable standardized process (see Figure 4).

The process is three-parted and contains the:

- Monitoring system/sensor module, which contains all services to monitor legacy systems and devices in enterprises vie the Data Access Layer. The distributed monitoring services also call back to this module with their gathered information. The monitoring services can be extended and configured for different data sources.
- Parser module, which contains content parser for the different possible data captured by the monitoring services. This module is parsing the content provided by the monitoring services so that it can be analyzed by the analyzer module. The parser can be extended and configured for different content provided by the monitoring services.
- Analyzer builder module, which correlates the monitored content and constructs the “Monitoring Data” to be stored and handed over to the Context Extractor or any other service that needs this information. The analyzer can be extended and configured for different content provided by the parser module.

### 5.3. Context Extractor

The objective of the Context Extraction is to extract and identify high-level context from the monitored data in the Context System Monitor. The service is based on a semantic model for an integrated representation of knowledge about devices, machines, manufacturing processes and environment.

**Figure 5:**
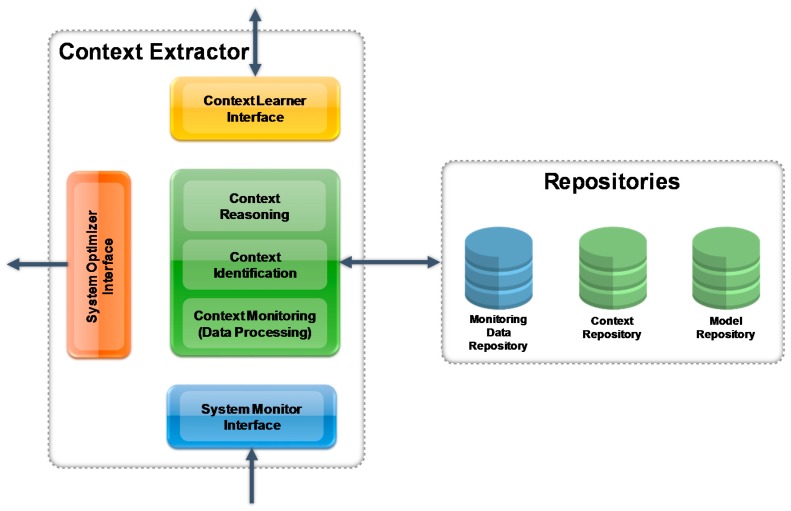
Context Extractor.

Figure 5 shows the Context Extractor Architecture. The Context Extractor tries to extract current context based on the monitored data provided by the System Monitor. The following process involves Context Identification and Context Reasoning. Finally, the extracted context published via the System Optimizer Interface. The corresponding modules comprise the following functionality:

- Context Model—see Section 5.1 above. All features in the Context Extractor are based on this model.
- Context Monitoring—see Section 5.2 above. The Context Monitoring acts as a proxy between System Monitor and Context Identification.
- Context Identification module, which analyses the Monitoring Data handed over by the Context System Monitor and extracts knowledge context such as what products or components are involved, what resources are used, and what items, parts or units are referenced or manipulated in the current on-going context (see the text to follow).
- Context reasoning module, a rule based system which reasons on the context provided by the Context Identification module, and refines current identified contexts. This module also compares the similarity between the current on-going contexts and historical contexts in the model repository.
- System optimizer interface, which provides the results of the context extraction modules to other up-stream modules/services.

#### 5.3.1. Context Identification

The Monitoring Data sent from the Context System Monitoring is analyzed by the Context Extraction to identify meaningful context concepts. The Monitoring Data contains only low level crowded data regarding context concepts. For example, product ID which is currently produced at the manufacturing system, temperature etc. These may not be meaningful context concepts yet according to the Context model. These data are transformed to the information through the conceptual structure of the Context Model (ontology), such as what concepts are being used, what resources (sensors, CPS) are involved etc. The process includes simple tasks such as mapping from the Monitoring Data format to the Context Model (Ontology) format, or more complex tasks such as querying all available information regarding a concept (e.g., product) to determine if it is an existing concept in the Model.

**Figure 6:**
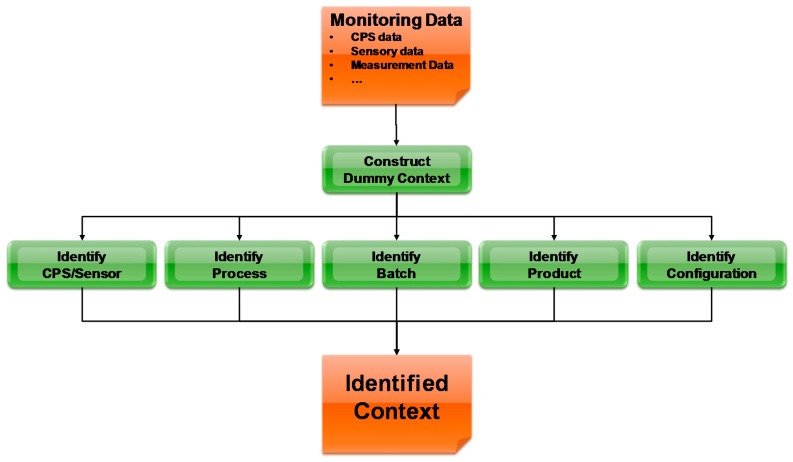
The Process of Context Identification.

As shown in Figure 6, the Context Identification process includes steps of identification of CPS, identifying process from sensors and CPS data, identifying production orders and produce items and resources, and constructing a dummy context to glue all identified data into the concepts included in the Context Model. This process is mainly realized by querying and mapping. As the monitoring data is represented in RDF format, SPARQL queries are issued to retrieve available information from it and to check with the Context Model, which is also represented in RDF. Then, based on the results, a mapping is implemented to generate high level context concepts into the Context Model by RDF manipulation.

#### 5.3.2. Context Reasoning

As it is often not possible to identify directly from the Monitoring Data high level context concepts included in the Context Model, the so-called Context Reasoning is applied allowing, based on diverse information from CPS and other sensors, such as the resources used, the actors involved as well as information of history contexts, to find out the context concepts values under which the system is currently working. Three types of reasoning technologies are used:

- Ontological Reasoning: based on the semantics of the ontology language and the definitions in the Context Model ontology the deductive reasoning is carried out, such as transitive reasoning and subsumption.
- Rule-based Reasoning: uses the deductive techniques as in the Ontological Context Reasoning, but with application-specific rules provided by users. Such application- or domain-specific rules could be provided by a domain expert or constructed based on a statistical analysis of historical data.
- Statistical Reasoning: does not rely on strict logical rules but instead tries to correlate information into possible relations, as suggested by the empirical data, to determine the most possible current context.

The Rule based Reasoning allows the identification of the context concepts by applying domain specific rules which use the data from current sensors. Under sensors are understood simple sensors (e.g., temperature, pressure), or complex sensors, including CPS treated as complex, intelligent sensors (e.g., a machine providing data on its current state etc.). The Context Identification uses various types of rules, the simplest one in a form:

(1) $$IF\;(\overset{n}{\underset{1}{\sum }}w_{i}sensor_{i}TRUE)\geq m_{j}\;THEN\;concept_{j}\;IS\;TRUE$$

where

- *sensor_i_ TRUE* has value 1 or 0 depending is the value of the current signal at *sensor i* (e.g., temperature) satisfying the sub-rules associated to the *sensor_i_* or not,
- *n* is the number of sensors relevant for the definition of concept *j*
- *w_i_* is the weighing of the sensor value in identification of the context concept *j*,
- *m_j_* is the margin to claim that the context concept *j* is true or not

For example, the rule can be:

IF *sensor*_1_ true (a processing device has a valve attached to it), +
*sensor*_2_ true (the valve is observed by pressure sensor which provides a resource identified as pressure in Bar) >2 *THEN*
“The processing device is of type Mixing-Head” (*w* is 1 for both sensors in this case).

This is the simple rule when the context is defined by one of the concepts in the context model (described above). In the more general case, the context is defined as a set of concepts in the context model and the rules are correspondingly more complex.

#### 5.3.3. Context Similarity Measurement

The Context Similarity Measure is applied in both Context Extractor (Context Reasoning) but also in Optimizer for Context-aware optimizations and selections. Basically, it compares two given contexts (concerning their related concepts regarding e.g., devices, equipment, processes etc.) by using the context hierarchy tree defined in the Context Model., to tell how similar they are [21].

A context situation is characterized by a context instance and its related context resources—concepts such as devices, units, processes etc. A context situation C can be defined as a set of context elements—($E_{i}$), i.e., a set of concepts in the Context Model which are relevant for the context situation. The following notions are defined:

- Current Context (CC): The Context (current situation) for which similarity with the context situations (cases) already stored in the database will be computed.
- Stored Context (SC): any of the previously stored context cases which are stored in the database, used for computing the similarity with CC.
- Raw Similarity (RS): the value of the absolute similarity between two entities before being weighted.
- Weighted Similarity (WS): the relative, i.e., weighted value of the similarity. The following equality stands: WS = RS * weight.

Then the similarity between two context situations $C_{1}$ and $C_{2}$ can be derived into similarity measures of these 2 elements, as shown in Equation (2):

(2) $$WS\left(CC,SC\right)=\overset{n}{\underset{1}{\sum }}w_{i}sim_{E_{i}}(E_{i}\left(CC\right),E_{i}\left(SC\right))$$

where $w_{i}$ is the weight of the element set, $\overset{n}{\underset{1}{\sum }}w_{i}=1$, $sim_{E_{i}}\left(E_{i}\left(\mathrm{CC}\right),E_{i}\left(\mathrm{SC}\right)\right)$ is the similarity between two element sets of CC and SC (the value is a real number between 0 and 1), n is the number of concepts involved in the context situation. The weight of each context element can be the same in a simple model, or a higher weight can be applied to the more important element in a more complex model. In the latter case, a domain expert or a group of experts have to define initial weights and a learning algorithm is implemented to adjust the weights based on user feedback.

The similarity between two element sets $sim_{E_{i}}\left(E_{i}\left(CC_{1}\right),\;E_{i}\left(SC_{2}\right)\right)$ is computed by exploiting the hierarchical class tree of an ontology as presented in [21].

### 5.4. Optimizer and Self-Learning

The Optimizer module includes services which update/optimize the system behavior based on the identified change of context. The Context Extractor identifies in run time the change in context and provides the information on the Optimizer. The optimizer then adapts the system behavior to the changed ‘conditions’. In the currently developed version of the Optimizer, it includes sets of rules to adapt the system behavior to the change of context. The rules are continuously updated by the Self-learning services which ‘learn’ how to improve the system behavior based on the user validation of the proposals made by the Optimizer. The domain specific rules are applied, as explained in Section 5.3. These rules are updated by the Self-learning services (weights in the rules) However, besides such self-learning rule based solution, the Optimizer may include different other solutions such as classical optimization algorithms etc.

Self-learning services are used during the Adaptation process, during the Proactive behavior and during Context Extraction. When an optimization process is triggered, the monitoring data are retrieved and encapsulated into a structure that in turn is sent to the learning module to be processed. The result is again encapsulated into a structure. The selected algorithm is instantiated, to be used for current optimization process as presented in [40]. Based on the input structure the learning module is able to instantiate the necessary number of algorithms to face the current application scenario.

Two core operations learn and reason, allow the training of the algorithm using a particular model and elaborating a result. The instantiated learning algorithms need a number of models to be trained, depending on the specific application. In the same application, several models can be used depending on the current application scenario. The number of instantiated algorithms determines the number of necessary models to use (see also [40]). An update of the existing learning models is carried out using the last optimization process result. The updated learning models serve to update the context model accordingly. The self-learning services are using then these new updated learning models, while the Context Extractor uses the updated context model.

The Learning Services have been implemented using RapidMiner [41]. This framework allows the easy integration and usage of several learning algorithms and is furthermore extensible. The following learning algorithms are possible to be executed through the Learning Services and are automatically selected based on the application requirements: ID3 Learner, Naïve-Bayes Learner, Support Vector Machine, Neural Networks, Rule Induction and Least Mean Square. The architecture of the Learning Services allows for an easy extension of existing learning algorithms as well as for integration of additional learning algorithms.

## 6. Experimental Results

As indicated above, the proposed solution is applicable to wide scope of systems. Two specific applications were investigated in practice, i.e., the above described solution is applied in two very different run-time optimizations. The adjustment of the generic solution to the specific applications includes: (a) definition/update of the context model relevant for the specific optimization and process; (b) definition of CPS and other sources of information as well updates of the rules to process these information needed for context extraction; (c) adjustment of the Optimizer initial rules to specific optimization.

### 6.1. Energy Consumption Optimization

The first scenario is the application of the proposed solution for the optimization of so-called secondary processes, in this case optimization of energy consumption of CNC machines. The above proposed solution is integrated to the existing service platform. The goal in this experiment is to improve machine tool energy consumption by using context aware and self-adapting solution, as an alternative to the common time-out strategy for reducing energy consumption (see also in [42]). The majority of the current procedures for optimization of energy consumptions of CNC machines require setting of time out in advance which leads to suboptimal energy consumption. The proposed solution aims to avoid this shortcoming of the current procedures.

**Figure 7:**
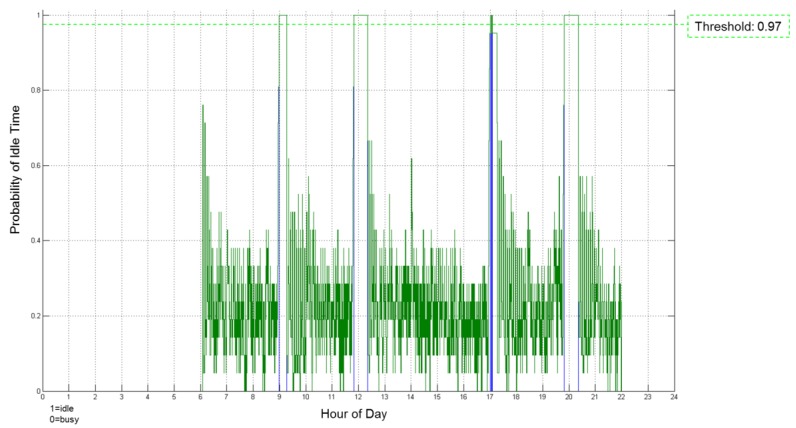
Example of detected machine idle times.

The selected Context Model includes specific extension of the generic model that are used to represent IDLE times. For IDL times concepts such as VTodo, VEvent, VAlarm and VFreeBusy are used. The actual machine being idle is identified and represented within the default generic context model as either a GenericDevice or some of its subclasses (like ProcessingDevice or ProductionUnit). The Context Extractor monitors several machine control states in run-time of the manufacturing process and identifies the idle time patterns in different time domains. The Context Extractor receives raw-data via the Data Access Layer and identifies the idle times, i.e., deduces high-level information from the received low-level raw data and checks the context consistency and reliability as well. Context Extractor encapsulates all identified machine idle times in a standardized meta-data model and notifies the Optimizer that the context has changed. The simple rules and the context similarity measure, as the one presented in Section 5.3, are applied. The machine tool data are classified in time domain (see Figure 7).

The identified idle times are sent to the Optimizer which proposes possible scheduling for energy saving tasks to be executed during the identified idle-times taking into account both their duration and the entire lifecycle of the machines and processes, i.e., taking into account the tasks executed in the past and various information describing the conditions under which the process is operating. The observation of the context, based on the data from CPS in processes, under which the machines/process are executed allows for modelling of machine tool behavior. This in turn allows to predict how the machine will be used in future and by this avoid setting of time-out, i.e., the machine can be shut-off once an idle time identified. Assuming that the characteristic wake up delay is known, the machine can be turned on in advance, and by this avoid delays and potential losses in productivity of the processes. In addition, the model of the machine behavior gained allows identification of the most appropriate energetic states for the context under which the machine is operating.

**Figure 8:**
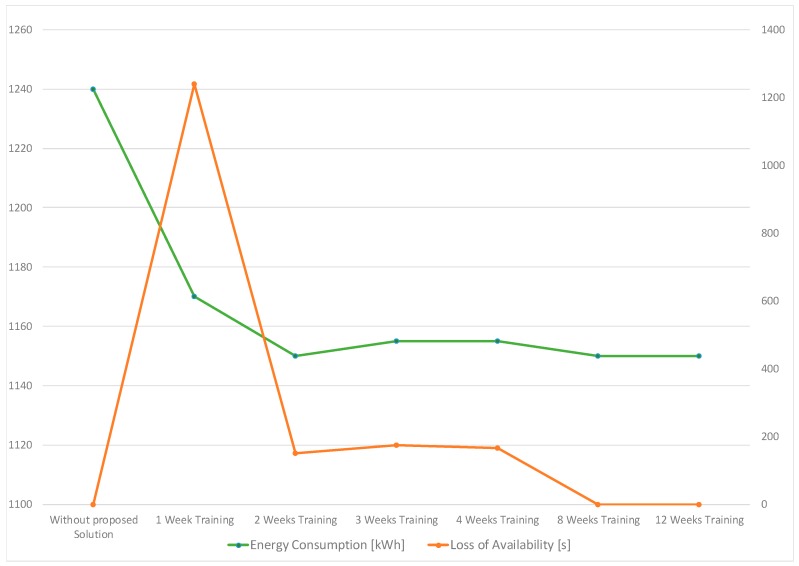
Energy saving result and machine availability.

The proposed solution has been tested on data gathered from real machine installations. The context sensitive solution was fed with the provided data and by this the capability of the solution to recognize the idle times patterns and schedule machine energy saving tasks has been explored. The selected energy saving tasks were then communicated to the shop floor machines using an OPC-UA connection. The solution has been tested over a period of time showing good levels of reliability, feasibility and robustness. The results concerning the energy savings achieved as well as the loss of machine availability occurring by using the proposed solution are shown in Figure 8.

It can be seen that the application of the proposed solution results in a considerable improvement in energy savings for machine tools. The baseline for comparison is the current practice in industry where, as indicated above, it is required to set time out in advance which in turn leads to suboptimal energy consumption. However, the presence of an initial transient phase where the energy saving improves while the machine availability decreases can be seen. This can be attributed to the learning model of the machine that initially has not enough entries to correctly predict machine behavior. As learning approaches ID3 Learner and Naïve-Bayes Learner have been applied, both providing similar results. After this transient phase, the system stabilizes, i.e., the Optimizer learns based on the expert decisions along time and populates the learning model of the machine with new entries enhancing its capability to generalize.

As a result, the machine availability loss reduces along time approaching zero. Furthermore, the loss of availability along time goes to zero.

### 6.2. Availability and Efficiency Optimization at CPS Based FMS

This experiment involved CPS based FMS in shoe industry. The production and manufacturing of shoes involves a wide variety of materials and a large number of operations. Such FMS comprise a set of complex operations that are labor intensive and are very dependent on the operator’s skill. The need for automatic recognition of current situations and continuous optimization of processes has been identified by the producers of FMS for shoe industry.

The proposed solution has been integrated into real industrial equipment. The Context Extractor identifies current context of production process and reacts to changing of context caused by variations in different parameter sets in order to improve error-prone processes (caused by humans) and reduce maintenance problems.

The selected experiment deals with a scenario in which the valves of a so called “mixing head” system shall be automatically adjusted based on the identified changing context. During the production process of shoe sole, different components are mixed by synchronously acting on different non-mechanical connected valves. The problem that arises after a vaguely defined time of shoe sole production, the valves get asynchronous. The influences that are causing the asynchronous operation of the valves are very different. Some of the cause are: different force requirements (due to changing products), different air supply, valve abrasion or operator’s skills. All these skills influence the quality of the final product. In the experiment the tested solution is continuously fed with manufacturing process parameters, which are collected from various CPS in the manufacturing process. The Context Model includes concepts, such as MixingHead, Tank, Material, SensoricalValveInformation, SensoricalTankInformation, SensoricalAmbitentTemperature-Information, SensoricalAmbitentHumidityInformation. In addition, several supporting concepts such as RotationsPerMinute, TimedCycle, Counter, FillingLevel have been introduced in the context model. These sets of parameters are used by the Optimizer to build a representative model of process relying on empirical data using data mining techniques. The parameters considered to build the model are the pressure and the temperature, speed frequencies of drives and pumps, proper material mix ratio and filling of materials into shoe forms etc.

The Context Extractor continuously tries to identify changing contexts. In case a changed context is identified, this context is sent to the Optimizer. The Optimizer starts an adaptation process, which results in a proposal for a set of production parameters to be changed. These Adaption proposal is evaluated by a human operator. Based on the results of adaptation and operator’s feedback, the learning services learn how changing cycle times and ambient conditions are influencing the production process (e.g., above explained valve synchronization) and update the rules for context identification, adaptation and extension. As learning algorithms, again ID3 Learner and Naïve-Bayes Learner have been applied and both provided similarly good results, while the application of the Support Vector Machine algorithm has not provided satisfactory results.

**Figure 9:**
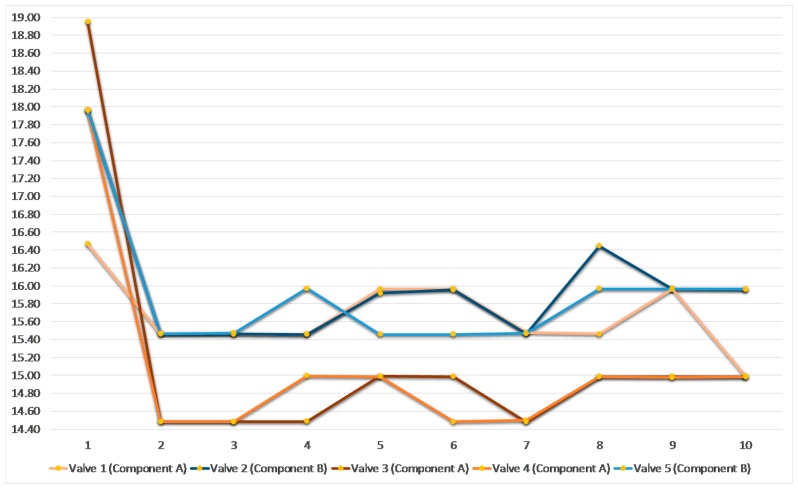
Results of a Valve Sync. test run (initial experiment) [4].

The prototype solution was tested in a demonstration set up of a real production environment. The results of the initial experiment for automatic valve synchronization are shown in Figure 9 (see also [4]). It is shown, that the opening times of the five valves that are used for injecting two types of materials (three valves for material A, two valves for material B) are continuously adapted to assure an “optimum” working range.

**Figure 10:**
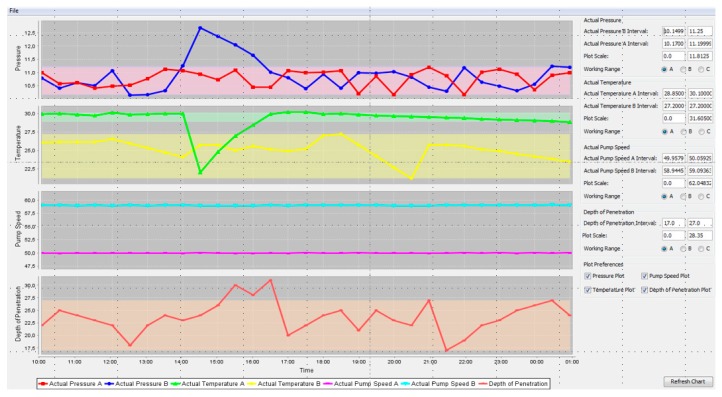
Results of advanced Valve Synchronization test run.

As it can be seen in the Figure 8, the “optimal” adjustments are achieved after an initial training phase. In further experiments the context model was extended to take into account additional machine/process parameters, such as Material Temperature, Pressure, Pump Speed, etc. The results of the “advanced” valve synchronization experiment (taking into account more input parameters) are shown in Figure 10 and confirm the results of the initial experiment. Both experiments demonstrate considerable improvements with respect to current practice in the industrial sector.

The experiments have proven that the proposed solution when applied in control of FMS in shoe industry may assure keeping of the process parameters inside the optimum working range under wide spectrum of changes in conditions under which FMS operate. Applying the proposed solution for self-adaptation of machine parameters, leads to an increased efficiency and availability, i.e., it may assure high utilization of machines and the product quality. Thereby changing ambient conditions are taken into account (identified changes in context).

## 7. Conclusions

The research presented in the paper resulted in an innovative context sensitivity solution to support run-time optimization of a wide scope of FMSs using run time information from various sensors and CPS, as complex intelligent sensor systems. The main benefit of the proposed generic solution is that it is easily adaptable to specific conditions of each system. The applicability of the solution for optimization of various parameters in two different manufacturing systems is demonstrated. The generic innovative context model has been proposed.

The building and maintenance of the optimization solutions in both applications using the proposed approach is considerably more effective than building classical scattered solutions. Contrary to e.g., classical adaptive control solutions, the generic solution bringing intelligence into the manufacturing processes, are easily applicable to various machines/processes, gaining a higher benefit for the manufacturer:

- The time/efforts spent for building the both above described applications is estimated to be more than 60% less than time/efforts needed to build individual solution for each optimization.
- The biggest advantages are seen in maintenance and extensibility of the solution: if the processes and conditions change (which is often in FMS processes) the solution can be easily maintained/updated by extending the context model and perhaps adding/modifying certain monitoring services and rules for better context extraction and adaptation, but the overall structure of the optimization solution needs not to be changed. It is estimated that the costs for maintenance of such solution is more than 80% lower than for maintenance of classical solutions.
- The benefit is that the proposed solution can be applied for a number (all) of optimization processes within a factory, i.e., the company does not need to apply a high number of various solutions, which in turn may radically reduce development and maintenance costs of such solutions.

New applications of such approach for run-time optimization in FMS are elaborated. Many research problems, however, are still under consideration. The decisions about which raw data are worthy to on-line collect/provide by monitoring services (which means efforts/costs to integrate services with various systems that hold these data) in order to better extract the context and support optimization, have to be made on case basis and are specific for each application. The proposed approach shall be integrated in the emerging Reference Architecture for Industry 4.0—RAMI 4.0 [43]. The methodology on how to analyze cost/benefit ratio for various applications is developed. The more complex rule based solutions, such as probabilistic reasoning and diverse learning algorithms have to be further explored experimentally. The key research issues to be solved are how to refine the context models. Automatic update of the context model based on the observed changes in environment is a subject of the further research. Another problem under study is how to assure better automatic evaluation and validation of the results to make learning process more autonomous.

It is likely that the proposed context sensitivity and self-learning approach, based on the advanced CPS as complex, intelligent sensors, providing information not available up to now and needed for context sensitivity, can be effectively applied in various application domains (e.g., logistics, health etc.).
